# Changes in soil organic matter over 70 years in continuous arable and ley–arable rotations on a sandy loam soil in England

**DOI:** 10.1111/ejss.12415

**Published:** 2017-03-21

**Authors:** A. E. Johnston, P. R. Poulton, K. Coleman, A. J. Macdonald, R. P. White

**Affiliations:** ^1^ Department of Sustainable Soils and Grassland SystemsRothamsted ResearchWest CommonHarpendenAL5 2JQUK; ^2^ Department of Computational and Systems BiologyRothamsted ResearchWest CommonHarpendenAL5 2JQUK

## Abstract

The sequestration in soil of organic carbon (SOC) derived from atmospheric carbon dioxide (CO_2_) by replacing arable crops with leys, has been measured over 70 years on a sandy loam soil. The experiment was designed initially to test the effect of leys on the yields of arable crops. A 3‐year grazed grass with clover (grass + clover) ley in a 5‐year rotation with arable crops increased percentage organic carbon (%OC) in the top 25 cm of the soil from 0.98 to 1.23 in 28 years, but with little further increase during the next 40 years with all‐grass leys given fertilizer nitrogen (N). In this second period, OC inputs were balanced by losses, suggesting that about 1.3% OC might be near the equilibrium content for this rotation. Including 3‐year lucerne (Medicago sativa) leys had little effect on %OC over 28 years, but after changing to grass + clover leys, %OC increased to 1.24 during the next 40 years. Eight‐year leys (all grass with N or grass + clover) in 10‐year rotations with arable crops were started in the 1970s, and after three rotations %OC had increased to ca. 1.40 in 2000–2009. Over 70 years, %OC declined from 0.98 to 0.94 in an all‐arable rotation with mainly cereals and to 0.82 with more root crops. Applications of 38 t ha^−1^ farmyard manure (FYM) every fifth year increased %OC by 0.13% by the mid‐1960s when applications ceased. Soil treated with FYM still contained 0.10% more OC in 2000–2009. Changes in the amount of OC have been modelled with RothC‐26.3 and estimated inputs of C for selected rotations. Little of the OC input during the 70 years has been retained; most was retained in the grazed ley rotation, but 9 t ha^−1^ only of a total input of 189 t ha^−1^. In other rotations more than 98% of the total OC input was lost. Despite large losses of C, annual increases in OC of 4‰ are possible on this soil type with the inclusion of grass or grass + clover leys or the application of FYM, but only for a limited period. Such increases in SOC might help to limit increases in atmospheric CO_2_.

**Highlights:**

- Can leys sequester significant amounts of atmospheric CO_2_ in SOM and contribute to the 4‰ initiative?
- Changes in the percentage and amount of OC were measured and modelled over 70 years and OC losses estimated.
- Three‐year grass or grass + clover leys increased %OC, but only to an equilibrium level that was then maintained.
- Despite large losses, sequestering CO_2_‐C at 4‰ year^−1^ by growing grass or grass + clover leys is possible.

## Introduction

One possible mitigation strategy, suggested by Soussana *et al*. (2007, 2010) and promoted by Le Foll (2015), to deal with the increase in concentration of carbon dioxide (CO_2_) in the atmosphere, could be to sequester more carbon (C) in soil organic matter (SOM). On agricultural land this could be carried out by changing cropping patterns to include more herbacious plants (Powlson *et al*., 2012 *;* Stockmann *et al*., 2013; Koch *et al*., 2015 *;* Chambers *et al*., 2016; Paustian *et al*., 2016). The Woburn ley–arable experiment (Bedfordshire, United Kingdom), which started in 1938 on a sandy loam soil with little SOM, was established to test whether growing more leys in mainly arable cropping systems would increase the yields of arable crops. Percentage organic carbon (%OC) in the topsoil was measured in 1938 and has been measured for each treatment every fifth year since the mid‐1950s. Thus, over this 70‐year period it is possible to assess how the amount of OC stored in the topsoil has changed. Two important issues are also illustrated: (i) in temperate climates amounts of OC change slowly in most cropping systems, and the amount depends on the input of added organic matter and the rate of decomposition of added and existing organic matter, soil type and climate (Johnston *et al*., 2009; Smith, 2014; White & Davidson, 2016) and (ii) for any one cropping system and soil type, the annual input of organic carbon (OC) in organic material is eventually balanced by the loss from the soil and consequently %OC changes little. An ‘equilibrium’ or ‘quasi‐equilibrium’ will be reached when %OC does not change, although the amount of OC in each pool of soil OC will be changing constantly. For a specific farming system, the equilibrium value of %OC is larger in a clay than in a sandy soil, and on any one soil type is larger under permanent grass than under continuous arable cropping (Johnston *et al*., 2009).

This paper reports changes over 70 years in both %OC and the amount of OC in the top 25 cm of a sandy loam soil under continuous arable rotations and ley–arable rotations with different types and lengths of ley. Farmyard manure (FYM) was applied once in 5 years to half the plots until the mid‐1960s, and its initial and residual effect on %OC is given. Changes in %OC indicate the direction of change when ley–arable rotations are compared with all‐arable rotations, and the amount of OC in soil has been calculated for different periods. For four of the rotations the amounts of OC added and lost have been estimated with the Rothamsted SOM turnover model, RothC‐26.3 (Jenkinson, 1990; Jenkinson *et al*., 1994). The estimated amount of OC retained is compared with that calculated from the change in %OC. The measured and modelled changes in OC are also discussed in relation to the 4‰ initiative proposed by Soussana *et al*. (2007, 2010) and Le Foll (2015), who contend that even modest increases in soil organic carbon (SOC) will help to limit increases in atmospheric CO_2_ and thus global temperature.

## Materials and methods

More leys (i.e. grasses, clovers or forage legumes such as lucerne (*Medicago sativa*) in arable crop rotations) were proposed in the 1930s to improve soil fertility. In addition, FYM, produced when the animals were housed overwinter, could be applied to fields with arable crops. Ley–arable and all‐arable cropping systems were tested and compared in England by Rothamsted Research (Boyd, 1968) and others (Eagle, 1975; Lowe, 1975). Only three Rothamsted experiments continue to the present. Overall changes in %OC in the two experiments at the Rothamsted farm (Harpenden, UK) are given in Johnston *et al*. (2009).

### Site and treatment details

The Woburn ley–arable experiment started in 1938 on a slightly sloping 1.6‐ha site that had grown arable crops since at least 1876 (Russell & Voelcker, 1936) and had 0.98% OC in the topsoil (Johnston, 1973). The soil is a sandy loam with 11–16% clay (Catt *et al*., 1980) and is classified as a Cambric Arenosol (FAO, 1990). The long‐term (1938–2007) average annual rainfall was 640 mm. Between 1938 and 1988 the average annual temperature was 9.3°C with a marginally downward trend, but since 1989 it has increased. The 5‐year mean for 2005–2009 was 10.4°C.

The experiment has five blocks so that all phases of the initial 5‐year rotations were present each year. Each block has eight pairs of plots; each plot is 8.5 m × 19.7 m. The experiment started with the first‐year treatment crops on Block III in 1938, and on Blocks V, IV, II and I in 1939, 1940, 1941 and 1942, respectively. The cropping history for Block III from 1938 to 2007 is given in Table 1. The cropping history for all five blocks is available from the Electronic Rothamsted Archive (e–RA; www.era.rothamsted.ac.uk). Changes in the arable crops and the composition of the leys were made to minimize the build‐up of pests and diseases and to maintain the continuation of the treatments (Johnston, 1997; Johnston & Poulton, 2005).

**Table 1:** Treatment crops and test crops^a^, 1938–2007, Block III^b^, Woburn ley–arable experiment

Year	Continuous rotations				Alternating rotations^c^ then 8‐year leys^d^			
	Arable		Ley–arable		First cycle		Second cycle	
	AB^e^	AF^f^	LN3^g^	LC3^h^	LN8^i^	LC8^j^	LN8^k^	LC8^l^
1938	P	P	L1	Lu1	P	P	Lu1	L1
1939	W	W	L2	Lu2	W	W	Lu2	L2
1940	H	K	L3	Lu3	K	H	Lu3	L3
1941	P	P	P	P	P	P	P	P
1942	B	B	B	B	B	B	B	B
1943	P	P	L1	Lu1	Lu1	L1	P	P
1944	W	W	L2	Lu2	Lu2	L2	W	W
1945	H	SBe	L3	Lu3	Lu3	L3	SBe	H
1946	P	P	P	P	P	P	P	P
1947	B	B	B	B	B	B	B	B
1948	P	P	L1	Lu1	P	P	L1	Lu1
1949	R	R	L2	Lu2	R	R	L2	Lu2
1950	H	SBe	L3	Lu3	H	SBe	L3	Lu3
1951	P	P	P	P	P	P	P	P
1952	B	B	B	B	B	B	B	B
1953	P	P	L1	Lu1	L1	Lu1	P	P
1954	R	R	L2	Lu2	L2	Lu2	R	R
1955	H	SBe	L3	Lu3	L3	Lu3	H	SBe
1956	SBe	SBe	SBe	SBe	SBe	SBe	SBe	SBe
1957	B	B	B	B	B	B	B	B
1958	P	P	L1	Lu1	P	P	Lu1	L1
1959	R	R	L2	Lu2	R	R	Lu2	L2
1960	H	C	L3	Lu3	H	C	Lu3	L3
1961	SBe	SBe	SBe	SBe	SBe	SBe	SBe	SBe
1962	B	B	B	B	B	B	B	B
1963	P	P	L1	Lu1	Lu1	L1	P	P
1964	R	R	L2	Lu2	Lu2	L2	R	R
1965	H	C	L3	S	S	L3	C	H
1966	SBe	SBe	SBe	SBe	SBe	SBe	SBe	SBe
1967	B	B	B	B	B	B	B	B
1968	P	P	L1	S1	P	P	L1	S1
1969	R	R	L2	S2	R	R	L2	S2
1970	H	C	L3	S3	C	H	L3	S3
1971	P	P	P	P	P	P	P	P
1972	W	W	W	W	W	W	W	W
1973	P	P	Ln1	Lc1	Ln1	Lc1	P	P
1974	B	B	Ln2	Lc2	Ln2	Lc2	B	B
1975	H	B	Ln3	Lc3	Ln3	Lc3	H	B
1976	W	W	W	W	Ln4	Lc4	W	W
1977	B	B	B	B	Ln5	Lc5	B	B
1978	B	F	Ln1	Lc1	Ln6	Lc6	Ln1	Lc1
1979	B	F	Ln2	Lc2	Ln7	Lc7	Ln2	Lc2
1980	O	O	Ln3	Lc3	Ln8	Lc8	Ln3	Lc3
1981	W	W	W	W	W	W	Ln4	Lc4
1982	B	B	B	B	B	B	Ln5	Lc5
1983	B	F	Ln1	Lc1	Ln1	Lc1	Ln6	Lc6
1984	B	F	Ln2	Lc2	Ln2	Lc2	Ln7	Lc7
1985	BE	BE	Ln3	Lc3	Ln3	Lc3	Ln8	Lc8
1986	W	W	W	W	Ln4	Lc4	W	W
1987	B	B	B	B	Ln5	Lc5	B	B
1988	B	F	Ln1	Lc1	Ln6	Lc6	Ln1	Lc1
1989	B	F	Ln2	Lc2	Ln7	Lc7	Ln2	Lc2
1990	BE	BE	Ln3	Lc3	Ln8	Lc8	Ln3	Lc3
1991	W	W	W	W	W	W	Ln4	Lc4
1992	R	R	R	R	R	R	Ln5	Lc5
1993	B	F	Ln1	Lc1	Ln1	Lc1	Ln6	Lc6
1994	B	F	Ln2	Lc2	Ln2	Lc2	Ln7	Lc7
1995	BE	BE	Ln3	Lc3	Ln3	Lc3	Ln8	Lc8
1996	W	W	W	W	Ln4	Lc4	W	W
1997	R	R	R	R	Ln5	Lc5	R	R
1998	R	R	Ln1	Lc1	Ln6	Lc6	Ln1	Lc1
1999	M	BE	Ln2	Lc2	Ln7	Lc7	Ln2	Lc2
2000	BE	M	Ln3	Lc3	Ln8	Lc8	Ln3	Lc3
2001	W	W	W	W	W	W	Ln4	Lc4
2002	R	R	R	R	R	R	Ln5	Lc5
2003	R	R	Ln1	Lc1	Ln1	Lc1	Ln6	Lc6
2004	M	BE	Ln2	Lc2	Ln2	Lc2	Ln7	Lc7
2005	BE	M	Ln3	Lc3	Ln3	Lc3	Ln8	Lc8
2006	W	W	W	W	Ln4	Lc4	W	W
2007	R	R	R	R	Ln5	Lc5	R	R

a Test crops are highlighted in grey. Plots were divided to test four rates of N when test crops were grown. The rates of N rotated so that, over time, the C inputs on the four subplots were similar.

b Treatment cropping started in 1938 on Block III, and in 1939, 1940, 1941 and 1942 on Blocks V, IV, II and I, respectively.

c On four pairs of plots treatment crops alternated between arable and ley rotations.

d The alternating rotations were replaced by 8‐year grass leys with N or 8‐year grass + clover leys. The first cycle of these longer leys started in 1973 on Block III and in 1974, 1975, 1976 and 1977 on Blocks V, IV, II and I, respectively. The second cycle of 8‐year leys started in 1978 on Block III and in 1979, 1980, 1981 and 1982 on Blocks V, IV, II and I, respectively. The delay in starting the second cycle of 8‐year leys meant that the effects of all of the different treatment rotations on the yield of the following test crops could be measured every 5 years.

e AB treatment crops: potatoes, cereal, 1‐year hay from 1938 to 1975; barley, barley, beans (or oats) from 1978 to 1995; rye, maize, beans since 1998.

f AF treatment crops: potatoes, cereal, root crop from 1938 to 1975; fallow, fallow, beans from 1978 to 1995; rye, beans, maize since 1998.

g LN3 treatment crop: 3‐year grazed grass + clover leys with N from 1938 to 1970; 3‐year grass leys with N since 1973.

h LC3 treatment crop: 3‐year lucerne or sainfoin leys from 1938 to 1970; 3‐year grass + clover leys since 1973.

i LN8 treatment crop: alternating treatment crops from 1938 to 1970; 8‐year grass leys with N since 1973 (first cycle).

j LC8 treatment crop: alternating treatment crops from 1938 to 1970; 8‐year grass + clover leys since 1973 (first cycle).

k LN8 treatment crop: alternating treatment crops from 1938 to 1975; 8‐year grass leys with N since 1978 (second cycle).

l LC8 treatment crop: alternating treatment crops from 1938 to 1975; 8‐year grass + clover leys since 1978 (second cycle).

P, potatoes; W, winter wheat; H, 1‐year hay; K, kale; B, spring barley; SBe, sugar beet; R, winter rye; C, carrots; F, fallow; O, winter oats; BE, winter beans; M, maize; L1–L3, first, second and third year of a grazed grass + clover ley; Lu1–Lu3, first, second and third year of a lucerne ley; S1–S3, first, second and third year of a sainfoin ley; Ln1–Ln8, first, second, third year etc. of a grass ley; Lc1–Lc8, first, second, third year etc. of a grass + clover ley.

Each of the eight rotations (AB, AF etc.) was grown on pairs of plots in each of five blocks. One plot in each pair received farmyard manure (FYM); 38 t ha^−1^ applied every fifth year to the first test crop of potatoes or sugar beet. Applications of FYM stopped when sugar beet was replaced as the first test crop in the mid‐1960s. The last applications of FYM were to Blocks IV, II, I, III and V in 1963, 1964, 1965, 1966 and 1967, respectively.

In this paper, recent codes are used for the rotation treatments: arable, AB and AF, and leys, LN3 and LC3 for the 5‐year rotations, and LN8 and LC8 for the 10‐year rotations; Table 1 shows how these relate to earlier and later cropping (and codes).

Initially, four pairs of plots were used to test four different 5‐year rotations (‘Continuous rotations’ on the left half of Table 1). The two ley–arable rotations (LN3 and LC3) had 3‐year leys (‘treatment’ crops) followed by two arable ‘test’ crops, and the two all‐arable rotations (AB and AF) had 3 years of arable ‘treatment’ crops followed by the same two arable ‘test’ crops. Yields of the ‘test’ crops measured the effect of growing either leys or arable crops in the preceding three ‘treatment’ years. Four rates of N were tested on the two test crops on four subplots in each plot. Initially, the leys were either grazed grass + clover (L) given a small amount (ca. 25 kg ha^−1^ year^−1^) of nitrogen (N), or lucerne (Lu) leys, which were replaced by sainfoin (*Onobrychis viciifoilia*) (S) leys for a few years. Further changes to the leys were phased in from 1972, starting with Block III (Table 1). Sainfoin was replaced by grass + clover (LC3) with 10–15% clover in the seed mixture, and the grazed ley (L) became an all‐grass ley (LN3) with 75 kg ha^−1^ fertilizer N for each cut. The leys are usually cut twice a year. The all‐arable rotations differed in the third treatment year only when either a 1‐year hay crop or a root crop was grown. The AF rotation was later changed to include 2 years of bare fallow.

The other four pairs of plots initially tested a complicated arrangement of alternating an all‐arable rotation with a ley–arable rotation (‘Alternating rotations’; right side of Table 1). These gave little useful information and were stopped in the 1970s and replaced by 10‐year rotations with 8‐year leys (either grass with N (LN8) or grass + clover (LC8)) followed by two arable crops (Table 1). These 8‐year leys were phased‐in in two cycles (see right side of Table 1) so that every 5 years the effects of continuous arable cropping and 3‐ and 8‐year leys on the yield of the following arable crops could be compared.

Initially, one plot in each of the eight pairs of plots received 38 t ha^−1^ FYM applied every fifth year to the first test crop of potatoes or sugar beet. These applications were stopped in the mid‐1960s, but this was not phased in the same way as most other changes. Therefore, FYM was applied five times on two blocks and six times on the other three (see footnote in Table 1).

Phosphorus (P) and potassium (K) are applied annually to maintain adequate levels of plant‐available P (Olsen P) and K (exchangeable K). Basal lime is applied to maintain topsoil pH at about 6.5–7.0. Treatment crops, except for lucerne, sainfoin and the later grass + clover (LC3 and LC8) leys, receive appropriate amounts of N. Four rates of N were tested on the two test crops on four subplots in each plot; these rates of N rotate on the subplots to ensure that the whole plot receives the same amount of N over time and therefore similar amounts of root and stubble residues. Above‐ground crop residues are removed after harvest, as is the green material on the ley plots.

### Soil sampling and analysis

Soil samples, 0–23 and 23–46 cm, were taken in March 1938 from each of the five blocks of the proposed experiment, and regular sampling started in the mid‐1950s, when each plot in the five blocks was sampled every fifth year to coincide with the end of the third treatment year or the eighth year of the 8‐year leys. For each rotation a 5‐ or 10‐year average for %OC can be calculated by averaging data from the five blocks. Soil samples were taken in a zig‐zag pattern from the central four‐fifths of the plot; most were from the top 25 cm of soil and consisted of 16–25 cores taken with a 2.5‐cm semi‐cylindrical sampler. The soil was then air‐dried in the laboratory. Soil %OC was determined on a ground (< 355 µm) subsample by the Tinsley method (Tinsley, 1950) before 1980; since then total soil carbon and total soil N have been determined by combustion (LECO Corp., St Joseph, Michigan, USA). Any inorganic carbon present as calcium carbonate, typically ca. 0.01% or less, was determined by manometry and subtracted from total carbon to give %OC. When the analytical technique changed, tests were carried out by our laboratory to ensure that data from the two techniques were compatible. For the few samples taken to 23 cm, %OC has been adjusted to a 25‐cm depth basis with an appropriate proportion of the %OC in the subsoil. Percentage OC can be converted to % soil organic matter by multiplying by the conventional factor, 1.72 (Mattingly, 1974). Topsoil was analysed for Olsen P, exchangeable K and Mg and pH to check that they would not limit crop yields. Analyses were carried out on ground (< 2 mm) subsamples.

### Weights of soil per hectare used to calculate amounts of soil organic carbon

When monitoring changes in the amount of carbon over time it is essential to sample the same weight of mineral soil on each occasion (Jenkinson *et al*., 2008), assuming that most SOM is held on the mineral particles. Where bulk density has not changed and, therefore, the weight of soil has not changed, the soil is sampled to the same depth each time. Where soil has been under pasture for some years, however, bulk density and hence the weight of soil at the depth originally sampled will have decreased. In this case, subsequent samples should be taken to a greater depth so that OC is measured on the same weight of mineral soil. This is rarely done but we have made an appropriate correction for the soil under leys in this experiment. On the same soil type, Mattingly (1974) measured the weight of soil for depths to 53 cm under arable crops and grass and grass + clover leys. From these we have calculated the weight of soil for the top 25 cm of 3770 t ha^−1^ for soil with continuous arable crops, and 3470 t ha^−1^ for soil where grass or grass + clover leys had been grown. The difference in soil weight, 300 t ha^−1^ (3770 minus 3470 t ha^−1^), represents the amount of ‘extra’ mineral soil that should have been sampled where leys had been grown. We have assumed that this additional 300 t ha^−1^ soil would have the same %OC as the top 25 cm soil in 1938 (i.e. 0.98 %OC) and would contain 2.9 t ha^−1^ OC on average for the five blocks. We have used the following weights of soil in the top 25 cm: for plots under continuous arable crops, 3770 t ha^−1^; for the LN3 plots from the mid‐1950s and LC3 plots from the mid‐1970s, and for the LN8 and LC8 plots from the mid‐1980s, 3470 t ha^−1^. To the amount of OC that we have calculated to be in the soil of the ley plots for the years shown, we have added 2.9 t ha^−1^ OC (i.e. the amount estimated to be in the ‘extra’ 300 t ha^−1^ soil that should have been sampled).

### Statistics

There is no replication in any year because each block is in a different phase of the rotation. The average %OC at the end of the third treatment year for each 5‐year rotation is the mean for that treatment in the five blocks (Table 2). For the 8‐year leys, %OC at the end of the third or eighth treatment year is the mean for all five blocks. Statistical analysis of the %OC data was carried out in two stages: first to assess the effects of the rotation and residual FYM treatments over each 5‐year cycle and subsequently to examine the trend in %OC over the duration of the experiment. The first used separate analyses of variance and the results are given in Table 2 with appropriate standard errors of differences. The trend was determined by regression over time. Departure from the linear response was modelled by superimposing splines that are different for the rotation and for the residual FYM treatments. The effects of the splines are considered random components in a residual maximum likelihood (REML) analysis, and tested by changes in deviance. Short‐ and longer‐term rotations were analysed separately for their trends. In the analyses for trend, the variable and factor associated with time is within each plot. The trends and associated errors are given in Table 3. Statistical analysis of the amount (t ha^−1^) of OC present in the topsoil used data from three periods, 1938, 1965–1974 and 2000–2009, to examine the effects of time and of the FYM residues and rotation treatments. The data for some treatments were adjusted to take account of the carbon contained in the ‘extra’ soil that should have been sampled (see above). The two factor interactions of time with each of the FYM residues and rotation treatments were statistically significant (i.e. the ‘with FYM residues’ responses are different from the ‘without FYM residues’ responses over time). Similarly, the rotation treatment responses with respect to time are significantly different from each other (see Table 4).

**Table 2:** Percentage of organic carbon in the top 25 cm of soil in different all‐arable and ley–arable cropping systems since 1938; mean of five blocks. Woburn ley–arable experiment

Rotation	FYM^a^ residues	Number of years since each block was phased in											
		0	18	23	28	33	38	43	48	53	58	63	68
		Years in which soils were sampled											
		1938	1955–9	1960–4	1965–9	1970–4	1975–9	1980–4	1985–9	1990–4	1995–9	2000–4	2005–9
AB	None or with	0.98	1.01	1.00	0.99	1.06	0.97	0.91	0.95	0.94	0.90	0.98	0.91
AF	None or with	0.98	0.95	0.93	0.93	0.94	0.90	0.82	0.82	0.82	0.75	0.84	0.80
LN3	None or with	0.98	1.14	1.17	1.23	1.32	1.19	1.20	1.19	1.22	1.13	1.31	1.27
LC3	None or with	0.98	1.06	1.02	1.04	1.10	1.10	1.08	1.13	1.18	1.16	1.24	1.24
Alternating rotations then long leys starting 1973–77 (first cycle)^b^													
LN8	None or with	0.98	1.06	1.04	1.07	1.09	(1.09)	1.13	(1.13)	1.29	(1.16)	1.47	(1.30)
LC8	None or with	0.98	1.06	1.03	1.08	1.12	(1.04)	1.12	(1.17)	1.22	(1.16)	1.36	(1.28)
Alternating rotations then long leys starting 1978–82 (second cycle)^c^													
LN8	None or with	0.98	1.02	0.98	0.97	1.04	1.02	(1.01)	1.14	(1.17)	1.18	(1.24)	1.38
LC8	None or with	0.98	1.01	1.08	1.08	1.12	1.07	(1.07)	1.17	(1.25)	1.18	(1.32)	1.43
All rotations	None	0.98	0.98	0.97	0.97	1.04	0.99	0.99	1.04	1.09	1.04	1.18	1.15
	With	0.98	1.09	1.10	1.12	1.16	1.11	1.10	1.13	1.18	1.12	1.26	1.25
Rotation	SED	—	0.061	0.057	0.054	0.067	0.065	0.066	0.069	0.08	0.073	0.074	0.095
	*F*‐ratio_(728)_	—	1.66	3.24	5.44	5.11	3.66	7.01	7.40	8.81	9.91	15.66	11.21
	*P*	—	n.s.	<0.05	<0.001	<0.001	<0.01	<0.001	<0.001	<0.001	<0.001	<0.001	<0.001
FYM residues	SED	—	0.017	0.015	0.018	0.014	0.016	0.015	0.02	0.019	0.015	0.019	0.021
	*F*‐ratio_(132)_	—	42.64	74.41	72.09	68.71	50.79	48.39	22.12	23.82	34.39	17.70	23.14
	*P*	—	<0.001	<0.001	<0.001	<0.001	<0.001	<0.001	<0.001	<0.001	<0.001	<0.001	<0.001

a See footnotes to Table 1 for dates of farmyard manure (FYM) applications.

b First cycle long leys were at the end of the eighth year of the 8‐year ley when sampled in 1980–1984, 1990–1994 and 2000–2004; data in brackets are at the end of the third year.

c Second cycle long leys were at the end of the eighth year of the 8‐year ley when sampled in 1985–1989 and 2005–2009; data in brackets are at the end of the third year.

AB, all‐arable rotation; AF, arable rotation with root crops or fallows; LN3, 3‐year grass leys + N followed by two arable crops; LC3, 3‐year grass + clover leys followed by two arable crops; LN8, 8‐year grass leys + N followed by two arable crops; LC8, 8‐year grass + clover leys followed by two arable crops; SED, standard error of differences of means.

**Table 3:** Summary of trends in percentage OC per 5‐year period; slopes of the fitted non‐linear trend lines and standard errors. Woburn ley–arable experiment

	Rotation			
	AB	AF	LN3	LC3
Slope	− 0.00861	− 0.01783	0.02167	0.01402
Standard error = 0.001719, *F*‐ratio_(4407)_ = 89.52, *P* < 0.001.				
Within each rotation there was no difference between with and without				
farmyard manure.				

	First and second cycle 8‐year leys	
	FYM	
	None	With
Slope	0.01865	0.02526
Standard error = 0.001691, *F*‐ratio_(2367)_ = 168.7, *P* < 0.001.		
There was no difference in the slope between the LN8 and LC8 rotations		
or the first and second cycles.		

AB, all‐arable rotation; AF, arable rotation with root crops or fallows; LN3, 3‐year grass leys + N followed by two arable crops; LC3, 3‐year grass + clover leys followed by two arable crops; LN8, 8‐year grass leys + N followed by two arable crops; LC8, 8‐year grass + clover leys followed by two arable crops.

**Table 4:** Total organic carbon, t ha^−1^, and rates of change, t ha^−1^ year^−1^, in the topsoil^a^ for different all‐arable and ley–arable cropping systems. Woburn ley–arable experiment

Rotation	FYM^b^ residues	Organic C / t ha^− 1^			Rate of change / t ha^− 1^ year^− 1^	
		1938	1965–74	2000–9	1938 to 1965–74	1965–74 to 2000–9
AB	None or with	36.9	38.7	35.5	0.06	−0.09
AF	None or with	36.9	35.2	31.0	−0.06	−0.12
LN3	None or with	36.9	47.1	47.6	0.33	0.01
LC3	None or with	36.9	40.3	45.9	0.11	0.16
LN8 c	None or with	36.9	39.7	52.3	0.09	0.36
LC8 c	None or with	36.9	41.4	51.3	0.15	0.28
All rotations	None	36.9	37.9	42.4	0.03	0.13
	With	36.9	42.9	45.5	0.20	0.07
Rotation × year group *F*‐ratio_(10 302)_ = 35.40, *P* < 0.001						
FYM × year group *F*‐ratio_(2302)_ = 15.01, *P* < 0.001.						
SED for all organic C = 1.84.						

a 0–25 cm, except where soil should have been sampled to a greater depth so that the same weight of soil was considered each time (see text).

b See footnotes to Table 1 for dates of farmyard manure (FYM) applications.

c For 2000–9, data for LN8 and LC8 are from the first (2000–4) and second cycles (2005–9); see Table 1.

AB, all‐arable rotation; AF, arable rotation with root crops or fallows; LN3, 3‐year grass leys + N followed by two arable crops; LC3, 3‐year grass + clover leys followed by two arable crops; LN8, 8‐year grass leys+N followed by two arable crops; LC8, 8‐year grass+clover leys followed by two arable crops; SED, standard error of differences of means.

### Data required to model the turnover of organic matter in soil

Changes in the amount of OC were modelled only for soil samples from the four 5‐year continuous rotations on Block III that did not receive FYM with the Rothamsted (RothC‐26.3) model for the turnover of organic matter in soil (Jenkinson, 1990; Jenkinson *et al*., 1994). A schematic diagram of this five‐compartment model is shown in Figure S1, Supporting Information. In addition to estimates of organic input, other information required to run the model included clay content (used to calculate water holding capacity and the proportion of CO_2_ that is evolved) and weather data (temperature, rainfall and evaporation) available from the meteorological station on Woburn Farm (www.era.rothamsted.ac.uk).

The input of organic matter was from roots, root exudates and stubble only because most above‐ground plant material was removed at harvest. In addition, but not allowed for, there were probably small inputs of organic matter from leaf litter where leys were grown. The inputs of C were calculated from the available yields (Anonymous, 1939–1986, 1987–2000, 2001–2009) following protocols from Bradbury *et al*. (1993), which relate estimates of below‐ground inputs to above‐ground yields, and with input estimates given by Mattingly (1974). Yields of the grazed leys from 1938 to 1970 were derived from the number of sheep‐grazing days and occasional yield measurements from which Mann & Boyd (1958) estimated the equivalent fresh yields. Yields of the leys from 1973 to 1983 were estimated from yields measured later. Yield data and our estimates of the annual C inputs from 1938 to 2007 are listed in Table S1, Supporting Information. We assumed that all the input C was in the topsoil (0–25 cm). The amount of carbon in the soil was obtained by running the RothC model with the estimated C inputs in Table S1 and the actual annual weather. At the start of the experiment the amount of carbon in the soil was estimated by running the model to equilibrium with an assumed C input of 1.74 t ha^−1^ year^−1^ and the known mean weather data for 1929–1938. From the estimates of carbon inputs and the amount remaining in the soil, the amount of OC lost as CO_2_‐C was calculated.

## Results and discussion

### Changes in the percentage of organic carbon in soil over 70 years of contrasted cropping

For each plot and sampling occasion, %OC and %N, when determined, are in e–RA (www.era.rothamsted.ac.uk). Changes in soil N are not discussed here. Changes in %OC that result from treatments are similar for all five blocks; therefore, the average of the five blocks is used throughout. In 1938 the site had been under arable cropping for about 100 years (Russell & Voelcker, 1936; Johnston, 1973) and samples taken from the top 25 cm of the soil in each of the five blocks had 0.98 %OC (range 0.89–1.11%).

**Figure 1:**
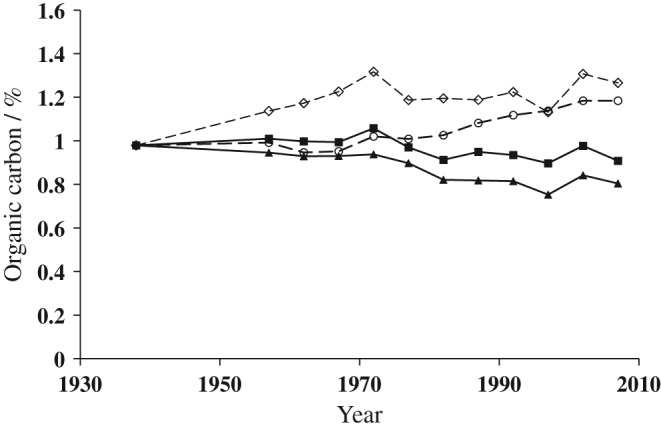
Percentage organic C in soil to 25 cm for selected treatments of the Woburn ley–arable experiment. Treatments are: (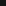) AB, all‐arable rotation; (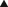) AF, arable rotation with root crops or fallows; (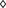) LN3, 3‐year grass leys + N followed by two arable crops; (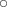) LC3, 3‐year grass + clover leys followed by two arable crops. See Table 1 for details of changes in cropping. Data are the mean of soil samples with or without farmyard manure (FYM); each point is the mean of data from each of the five blocks.

Values of %OC for each treatment from the mid‐1950s, averaged over soil with and without the addition of FYM, are given in Table 2. Significant differences between the rotations began to emerge in the early 1960s, more than 20 years after the experiment started. By 1960–1964, the soil with the 3‐year grazed ley (LN3) in each 5‐year rotation had significantly larger %OC than soil in the arable rotations. Following the change to a 3‐year grass ley given adequate N in the early 1970s, %OC was maintained at a significantly greater concentration than for the all‐arable rotations. Unexpectedly, the lucerne or sainfoin leys grown until the late 1960s did not increase %OC more than in the AB rotation. A similar result was seen in the Rothamsted ley–arable experiments (Johnston, 1973). At Woburn, the sainfoin leys were replaced by grass + clover leys (LC3) in the early 1970s, which resulted in an increase to 1.24 %OC by 2005–2009, which is similar to the 1.27 %OC in the LN3 soil. Both of these values are significantly greater than the %OC in AB and AF rotations (Table 2, Figure 1).

By 2005–2009, soil in the AB rotation contained 0.91 %OC and had changed little from the initial value of 0.98 %OC. In the AF rotation %OC declined from 0.98 to 0.80 (Table 2, Figure 1). The difference between the two arable rotations is not significant, but has been consistent throughout the 70 years and resulted from the differences in cropping. The AF rotation initially had two root crops in the three treatment years (Table 1), and then from the late 1970s to the late 1990s there were 2 years with fallow during the 3 years of treatment with no C input. Additional cultivation of the soil for root crops and fallow would increase soil aeration, which favours soil microbial decomposition of SOM. These changes in SOC over many years are similar to those in long‐term experiments with mainly arable crops at Askov in Denmark, also on a sandy loam soil (Christensen & Johnston, 1997).

In the early 1970s, %OC values in soil under alternating rotations (right side of Table 1) were similar to those in the AB rotation (Table 2). When these rotations were replaced by those with 8 years of grass (LN8) or grass + clover (LC8) leys (right side of Table 1), %OC increased and at the end of the third period of 8‐year leys (i.e. by 2000–2004 or 2005–2009 for the first and second cycle, respectively), the LN8 and LC8 soil contained 1.42 %OC and 1.40 %OC, respectively (means of first and second cycles), significantly greater concentrations than in the AB and AF rotations (Table 2).

The slopes of the fitted lines and associated errors for the trend in the long‐term changes in %OC for each of the four continuous 5‐year rotations are given in Table 3. The percentage of OC in both the AB and AF rotations declined significantly, whereas that in the LN3 and LC3 rotations increased significantly. For each of these four rotations there was no significant difference between soil with and without FYM.

### Changes in the percentage of carbon in soil with FYM

Although the increase in %OC in soil that received FYM once in 5 years until the mid‐1960s was very significant for each period when averaged over all rotations (Table 2), within each rotation the increase was not significant. In long‐term experiments without replication, however, consistency in the data and long‐term trends, which may not be statistically significant, are important. Table S2 (Supporting Information) shows that in the soil with continuous arable rotations the %OC had increased by ca. 0.10% in soil treated with FYM by the time applications stopped in the mid‐1960s, but there was a small difference only between soil with and without FYM residues in 2005–2009. Percentage OC increased more, by ca. 0.17%, with 3‐year leys (LN3 and LC3 rotations), and by 2005–2009 there was still a greater difference between soil with and without FYM residues than in the all‐arable rotations.

**Figure 2:**
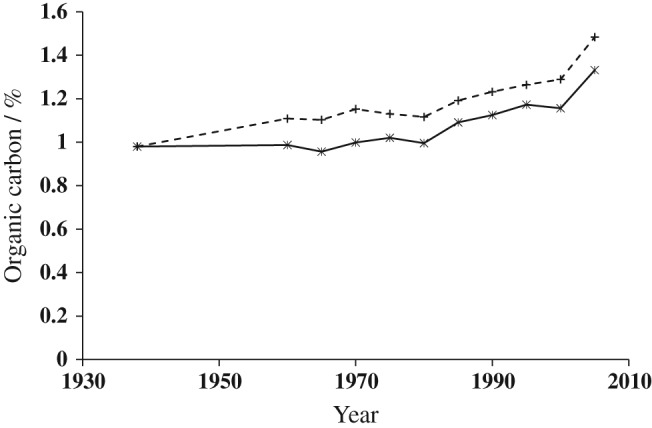
Percentage organic C in soil to 25 cm for treatments that changed to include 8‐year ley rotations in the Woburn ley–arable experiment. Data are the mean of the first and second cycles (brought into coincidence) for the 8‐year grass (LN8) or grass + clover (LC8) leys followed by two arable crops: without farmyard manure (FYM) (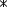) or with FYM (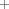). See Table 1 for details of changes in cropping. Each point is the mean of data from each of the five blocks.

The 8‐year leys were introduced after FYM applications ceased, but there was still a difference in %OC between soil with and without FYM residues in 2005–2009 (Table S2, Supporting Information) for both cycles of the 8‐year leys (first cycle, 1980–1984; second cycle, 1985–1989). When data for the two cycles were averaged (Table S2), but with the data from soil taken in the third year of an 8‐year ley disregarded (data in brackets in Table S2), there was a marked difference between the soil with and without FYM (Figure 2). Fitting separate trend lines to all of the LN8 and LC8 data showed that there was no difference between the types of ley, but that there was a significant difference in the slope of the fitted line between soil with and without FYM (Table 3).

The retention of more FYM‐C where leys were grown was probably because there were fewer cultivations, including inversion ploughing, during this period, resulting in less soil aeration, which would have decreased microbial decomposition of the added organic matter.

### Changes in the amount of organic carbon, t ha^−1^, during 70 years

Table 4 shows the amount of OC, t ha^−1^, in the topsoil in 1938 and 1965–1974 before major changes were made to the composition of the leys and 8‐year leys were introduced, and in 2000–2009 some 60–70 years after the experiment started. The 10‐year periods enable a direct comparison between 3‐ and 8‐year leys and the all‐arable rotations. Data for the 3‐year ley and arable rotations are the average of two consecutive 5‐year periods; for the 8‐year leys they are the average of the first and second cycles. The amount of OC in the soil has been calculated from the %OC and the weights of soil given earlier. For the LN3 soil in 1965–1974 and LN3, LC3, LN8 and LC8 in the final period, the additional OC was added as explained in the Section on *Materials and Methods*.

Over the 70‐year period there has been little change in the amount of OC in the all‐arable AB rotation (mean of without and with FYM residues; Table 4). In the AF rotation, which initially included more root crops and then several years without a crop (fallow), the amount of OC declined slowly by about 0.09 t ha^−1^ year^−1^ (Table 4). No straw or other above‐ground crop residues were ploughed in to either of the arable rotations. Machado (2011) also reported that the amount of OC in soil (0–60 cm) decreased where regular bare fallows were included in the rotation, but was maintained by applications of manure.

Compared with changes in the amount of OC in the all‐arable rotations, more OC accumulated where leys were grown, but the increases differed in relation to type of ley, its management and time. In the first period of the LN3 rotation, the ley of grass + clover was given a little fertilizer N and grazed by sheep, and OC increased significantly to 47.1 t ha^−1^ by 1965–1974 (ca. 0.33 t ha^−1^ year^−1^) compared with the all‐arable rotations. This amount was maintained but did not increase when the ley was changed to an all‐grass one with more fertilizer N (Table 4), presumably because SOM was at quasi‐equilibrium for this rotation. In contrast, lucerne or sainfoin leys did not result in a significant increase in the amount of OC. After this ley was changed to grass + clover (LC3) in the early 1970s, the amount of OC began to accumulate, and by 2000–2009 the soil contained 45.9 t ha^−1^, significantly more than in the all‐arable rotations (Table 4). Similarly, on soil where ley‐arable and all‐arable rotations initially alternated (Alternating rotations, Table 1), there was little or no increase in OC compared with the AB rotation, until the introduction of the 8‐year leys in the early 1970s. Since then, OC increased significantly, at a rate of 0.36 and 0.28 t ha^−1^ year^−1^ for the LN8 and LC8 soils, respectively (Table 4), compared with the AB rotation. The rates of increase in OC with the LN3 treatment during the first half of the experiment and with the LC3 and 8‐year leys in the later years are similar to those reported by Poeplau *et al*. (2015) for several ryegrass cover crop experiments (0.32 ± 0.28 t C ha^−1^ year^−1^), and by Post & Kwon (2000) for a wide range of soil types after being sown with grass (0.33 t C ha^−1^ year^−1^).

### Changes in the amount of carbon in soil where FYM was applied and retention of FYM‐derived carbon

A total of 212.8 t ha^−1^ FYM (average of plots that had received five or six applications; see footnote to Table 1) with an average dry matter content of 21.5% had been applied to the FYM‐treated plots when applications ceased. Assuming 40% C in dry matter, the total input of OC was 18.3 t ha^−1^. There was significantly more OC, 5.0 t ha^−1^, averaged over all rotations (42.9 minus 37.9 t OC ha^−1^), in the soil in 1965–1974 where FYM had been applied (Table 4), equivalent to ca. 27% of the carbon that had been added. By 2000–2009, an additional 3.1 t OC ha^−1^, ca. 17% of the amount added, was still in the soil that had last received FYM some 40 years previously (Table 4). The retention of 27% of added FYM‐C in this experiment is much less than that in an adjacent experiment where 250 t FYM ha^−1^ was applied annually over a much shorter period and where 50% of the FYM‐C was retained in the soil (Mattingly, 1974); this illustrates that the increase in OC is related to the amount added.

### Modelling changes in the amount of carbon retained in the soil and estimating losses

Changes in the amount of OC in four different rotations, AB, AF, LN3 and LC3, without FYM for Block III only were modelled with RothC‐26.3. Table S1(Supporting Information) gives the measured yields and estimated inputs of OC from 1938 to 2007; the inputs were similar to those given by Kuzyakov & Domanski (2000), Gill *et al*. (2002) and Wiesmeier *et al*. (2014). Table 5 lists the estimated total and average annual inputs of C for two periods, 1938–1970 and 1971–2007 (derived from Table S1); they range from ca. 1.4 to 2.8 t ha^−1^ year^−1^. Table 5 gives the modelled change in OC and the modelled loss of OC in t ha^−1^.

**Table 5:** Estimated inputs of C, t ha^−1^, and predicted changes in soil C, t ha^−1^, and losses of CO_2_‐C, t ha^−1^, over 69 years from the soil organic matter turnover model, RothC‐26.3; selected treatments, Block III. Woburn ley–arable experiment

Rotation	Estimated input of C / t ha^− 1^				Modelled change in soil C / t ha^− 1^				Modelled loss of CO_2_‐C / t ha^− 1^			
	1938–1970		1971–2007		1938–1970		1971–2007		1938–1970		1971–2007	
	Total	Annual	Total	Annual	Total	Annual	Total	Annual	Total	Annual	Total	Annual
AB	56.8	1.78	83.4	2.25	0.7	0.021	0.3	0.008	56.2	1.76	83.0	2.24
AF	58.1	1.82	63.6	1.72	0.3	0.011	−3.8	−0.102	57.7	1.80	67.4	1.82
LN3	89.3	2.79	99.4	2.68	8.4	0.263	0.2	0.006	80.9	2.53	99.1	2.68
LC3	45.0	1.41	89.2	2.41	−1.3	−0.041	4.1	0.111	46.3	1.45	85.0	2.30

AB, all‐arable rotation; AF, arable rotation with root crops or fallows; LN3, 3‐year grass leys + N followed by two arable crops; LC3, 3‐year grass + clover leys followed by two arable crops.

**Figure 3:**
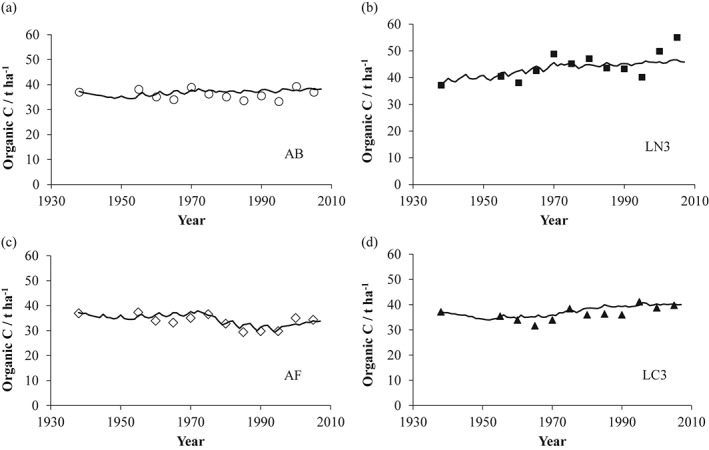
Organic C, t ha^−1^, in soil to 25 cm for selected treatments in Block III of the Woburn ley–arable experiment. The data points have been adjusted, where necessary, for changes in bulk density (see text) and the solid lines are the modelled output from RothC‐26.3. Treatments are: (a) AB, all‐arable rotation, (b) LN3, 3‐year grass leys + N followed by two arable crops, (c) AF, arable rotation with root crops or fallows and (d) LC3, 3‐year grass + clover leys followed by two arable crops. See Table 1 for details of changes in cropping. Data are from soil samples that did not receive farmyard manure (FYM). To obtain a modelled value for the amount of carbon in the soil at the start of the experiment a carbon input of 1.74 t ha^−1^ year^−1^ was used together with mean weather data for 1929–38. The soil was assumed to contain 3.0 t ha^−1^ of resistant organic matter (IOM).

Figure 3(a–d) shows that RothC‐26.3 simulated changes in the amount of OC well in this complicated experiment for the AB, AF and LC3 rotations but slightly less well for LN3, possibly because the OC inputs from grass leys were more variable from year to year than we estimated. For each of the four rotations the modelled estimates for the change in the amount of OC for soil without FYM in Block III (Table 5) broadly confirm the data based on the change in %OC (average with and without FYM for all five blocks) and appropriate weights of soil (Table 4). This strongly suggests that the modelled estimates for the input of OC and the loss of OC in Block III, most probably as CO_2_, given in Table 5 are representative of the whole experiment.

The estimated losses of OC in each rotation are large (Table 5) over the 70‐year period. For the all‐arable rotation of mainly cereals (AB rotation), the total estimated input of OC was 140 t ha^−1^. Most of the latter has been lost because there is little change in the amount of OC in the topsoil. In the AF rotation all of the OC input, 122 t ha^−1^, has been lost together with some existing soil OC, probably because the additional soil cultivations increased microbial decomposition of existing SOM. Since the mid‐1990s with cereals replacing fallow in this rotation (Table 1), the measured and modelled data have both shown a slight increase in the amount of OC (Figure 3c). Even in the LN3 and LC3 rotations with some increase in OC, losses have been large, 180 of the 189 t ha^−1^ input in the LN3 rotation and 131 of the 134 t ha^−1^ input in the LC3 rotation.

Table 5 gives the retention of the OC input in LN3 and LC3 rotations. Cropping in the LN3 rotation was in two periods. Between 1939 and 1970, when grazed grass + clover leys were given a little fertilizer N, the model predicts that about 9.4% of the OC added by 1970 was retained in the soil at an average annual rate of accumulation of 0.26 t ha^−1^ OC. Little more C was sequestered after 1971 when the ley was changed to all‐grass with adequate N (Table 5).

Cropping in the LC3 rotation was also in two periods. In the first period, yields of lucerne were often poor (Anon, a), and estimated OC inputs were less than with the arable crops (Table S1, Supporting Information) and SOC decreased until the early 1970s. Following the introduction of the grass + clover leys (Table 1), the model predicts that about 4.6% of the OC input was retained in the soil at an average annual rate of 0.11 t ha^−1^. In addition to the poorer yields of lucerne than of grass or clover, there is also a difference in root structure. Lucerne has a long taproot with few branches, whereas grass and clover both have more fibrous root systems growing mainly in the topsoil. Cooke & Williams (1972) found less than half as much root in the topsoil on a sandy clay loam after lucerne than after grass.

### Changes in soil organic carbon in this experiment in relation to the 4‰ initiative

The maximum annual rates of OC sequestration reported here, both measured and modelled, for soil in ley–arable rotations (0.26–0.36 t OC ha^−1^ year^−1^; Tables 4 5) are similar to those reported by Poeplau *et al*. (2015) and by Post & Kwon (2000). They are less, however, than the 1.1 t OC ha^−1^ year^−1^ reported by Christensen *et al*. (2009) for a 6‐year period of intensively managed grass, probably because our measurements encompassed years when arable crops were grown and some of the OC accumulated under the ley was lost when it was ploughed and cultivated for arable crops.

Our results can be considered in relation to the target proposed by Le Foll (2015) of an annual increase of 4‰ in soil OC to a depth of 40 cm to help mitigate further increases in global temperatures. The increases we measured are in the top 25 cm, but we have calculated them to 40 cm from the OC measured at the 23–46 cm depth in the soil in 1938 and we give this value in parenthesis.

The measured increase of 0.33 t OC ha^−1^ year^−1^ on the LN3 rotation in the first 28 years when the ley was grazed (Table 4) was an average from soil with and without FYM and is equivalent to an annual increase of 9‰ (6‰ for 0–40 cm). Although the grass leys grown in this rotation have maintained the OC content, there was no further significant increase in the amount over the next 40 years, which we suggest is because OC had reached an equilibrium for this soil and cropping system. Where no FYM was applied, the modelled data for this rotation (Table 5) suggest that OC increased by 0.26 t ha^−1^ year^−1^ (7‰ year^−1^) during the first 28 years (5‰ year^−1^ for 0–40 cm). Although it is possible to achieve an increase of 4‰ year^−1^ in soil OC to a depth of 40 cm by including leys in predominantly arable cropping rotations, it would be for a limited period only. For soil that changed to a 3‐year grass + clover ley or 8‐year all‐grass or grass + clover leys in the early 1970s, the increases in OC by 2000–2009 ranged from 4 to 9‰ year^−1^ (3–7‰ year^−1^ at 0–40 cm).

With FYM applied once every 5 years until the mid‐1960s and averaged over all rotations, OC increased by 0.17 t ha^−1^ year^−1^ compared with soil that did not receive FYM (Table 4), an increase of 5‰ year^−1^ (3‰ year^−1^ at 0–40 cm).

The increases in OC measured on our site, where grass or grass + clover leys have been grown in rotation with arable crops or where FYM has been applied, show that for this sandy soil type it is possible to sequester limited amounts of C from atmospheric CO_2_ in the SOC, but that much of the total C input will be lost. Once inputs are balanced by losses when a new equilibrium for soil OC has been reached, no further CO_2_‐C will be sequestered. It is also important to note that the inclusion of leys into any arable rotation has to be a commercially sensible option for the farmer. Any subsequent return to all‐arable cropping would lead to a decline in SOC.

## Conclusions

When the ley–arable experiment started in 1938 on a sandy loam soil the topsoil contained 0.98 %OC after growing arable crops for > 60 years. Where the soil remained in continuous arable cropping OC declined, more so where more root crops or fallows were included. The 3‐year grass or grass + clover leys in a 5‐year ley–arable rotation increased OC in the topsoil significantly over periods of 30–40 years, but lucerne or sainfoin leys did not. A concentration of about 1.3 %OC in the topsoil (0–25 cm) appears to be the equilibrium value where 3‐year grass or grass + clover leys were grown. Where the length of the leys was increased to 8 years in a 10‐year rotation with arable crops, %OC had not reached equilibrium after more than 30 years. Applications of FYM every fifth year also increased OC significantly. The results from modelling with RothC‐26.3 showed that, at most, 5% only of the added OC was retained in the soil and that 95% was lost. Measured and modelled changes in OC show that where grass or grass + clover leys were grown, annual increases of 4‰ or more are achievable, but for a limited period only until a new equilibrium for SOC is reached, and only while the OC inputs from the leys are maintained.

## Supporting information


**Table S1.** Yields, t ha^−1^, and estimates of carbon inputs, t ha^−1^, to the soil on selected plots without FYM residues, Block III only, Woburn ley–arable experiment.
**Table S2.** Percentage of organic carbon in the top 25 cm of soil with and without FYM residues, in different all‐arable and ley–arable cropping systems since 1938; mean of five blocks, Woburn ley–arable experiment.
**Figure S1.** Schematic diagram showing the flow of carbon through the Rothamsted turnover model (RothC‐26.3) with rate constants and turnover times for the different carbon pools. Redrawn, with permission, from Jenkinson et al. (1994).
