# Supplementary material for: Changes in soil organic matter over 70 years in continuous arable and ley–arable rotations on a sandy loam soil in England
Source: Eur J Soil Sci. 2017 Mar 21;68(3):305–16. doi: 10.1111/ejss.12415 (PMC5439491; doi:10.1111/ejss.12415)
Supplement: Supplementary file 1 — Table S1. Yields, t ha−1, and estimates of carbon inputs, t ha−1, to the soil on selected plots without FYM residues, Block III only, Woburn ley–arable experiment. Table S2. Percentage of organic carbon in the top 25 cm of soil with and without FYM residues, in different all‐arable and ley–arable cropping systems since 1938; mean of five blocks, Woburn ley–arable experiment. Figure S1. Schematic diagram showing the flow of carbon through the Rothamsted turnover model (RothC‐26.3) with rate constants and turnover times for the different carbon pools. Redrawn, with permission, from Jenkinson et al. (1994). [file EJSS-68-305-s001.doc]

**Table S1** Yields, , and estimates of carbon inputs, , to the soil on selected plots without FYM applications, Block III only. Woburn ley–arable experiment.

|  | AB^a^ | | | AF^b^ | | | LN3^c^ | | | LC3^d^ | | |
| --- | --- | --- | --- | --- | --- | --- | --- | --- | --- | --- | --- | --- |
| Year | Crop^e^ | Above ground yield^f^/ | Estimate of C input to the soil^g^/ | Crop | Above ground yield/ | Estimate of C input to the soil^g^/ | Crop | Above ground yield/ | Estimate of C input to the soil^g^/ | Crop | Above ground yield/ | Estimate of C input to the soil^g^/ |
| 1938 | P | 32.1 | 1.38 | P | 32.1 | 1.38 | L1 | 5.9 | 2.86 | Lu1 | 0.5 | 0.57 |
| 1939 | W | 1.6 | 1.46 | W | 1.6 | 1.46 | L2 | 10.1 | 3.40 | Lu2 | 1.3 | 0.80 |
| 1940 | H | 0.1 | 0.57 | K | 0.9 | 1.03 | L3 | 11.5 | 3.49 | Lu3 | 2.2 | 1.01 |
| 1941 | P | 29.7 | 1.33 | P | 23.5 | 1.18 | P | 38.1 | 1.48 | P | 28.0 | 1.29 |
| 1942 | B | 1.7 | 1.14 | B | 1.2 | 0.94 | B | 2.3 | 1.34 | B | 2.8 | 1.50 |
| 1943 | P | 21.9 | 1.14 | P | 17.8 | 1.03 | L1 | 5.9 | 2.86 | Lu1 | 0.3 | 0.50 |
| 1944 | W | 1.4 | 1.36 | W | 0.6 | 0.90 | L2 | 10.1 | 3.40 | Lu2 | 2.1 | 0.99 |
| 1945 | H | 0.2 | 0.64 | SBe | 17.4 | 2.55 | L3 | 11.5 | 3.49 | Lu3 | 2.8 | 1.14 |
| 1946 | P | 22.4 | 1.16 | P | 20.9 | 1.12 | P | 28.6 | 1.30 | P | 24.1 | 1.20 |
| 1947 | B | 1.5 | 1.06 | B | 1.3 | 0.98 | B | 1.9 | 1.21 | B | 1.6 | 1.10 |
| 1948 | P | 38.2 | 1.48 | P | 41.2 | 1.53 | L1 | 5.9 | 2.86 | Lu1 | 0.3 | 0.50 |
| 1949 | R | 3.4 | 1.97 | R | 3.3 | 1.96 | L2 | 10.1 | 3.40 | Lu2 | 1.9 | 0.95 |
| 1950 | H | 4.8 | 2.62 | SBe | 39.1 | 2.60 | L3 | 11.5 | 3.49 | Lu3 | 3.6 | 1.28 |
| 1951 | P | 27.1 | 1.27 | P | 27.2 | 1.27 | P | 37.0 | 1.46 | P | 31.7 | 1.37 |
| 1952 | B | 1.8 | 1.17 | B | 2.1 | 1.28 | B | 3.0 | 1.55 | B | 2.8 | 1.50 |
| 1953 | P | 31.0 | 1.35 | P | 28.3 | 1.30 | L1 | 5.9 | 2.86 | Lu | 4.6 | 1.42 |
| 1954 | R | 4.3 | 2.09 | R | 3.7 | 2.02 | L2 | 10.1 | 3.40 | Lu | 7.8 | 1.71 |
| 1955 | H | 6.8 | 3.02 | SBe | 29.8 | 2.60 | L3 | 11.5 | 3.49 | Lu | 6.8 | 1.64 |
| 1956 | SBe | 31.7 | 2.60 | SBe | 35.4 | 2.60 | SBe | 39.3 | 2.60 | SBe | 34.5 | 2.60 |
| 1957 | B | 4.1 | 1.81 | B | 3.8 | 1.75 | B | 4.2 | 1.83 | B | 4.2 | 1.83 |
| 1958 | P | 29.1 | 1.31 | P | 33.6 | 1.40 | L1 | 11.6 | 3.50 | Lu1 | 2.9 | 1.15 |
| 1959 | R | 3.8 | 2.03 | R | 3.5 | 1.99 | L2 | 7.4 | 3.11 | Lu2 | 4.1 | 1.35 |
| 1960 | H | 4.7 | 2.59 | C | 19.7 | 3.02 | L3 | 9.8 | 3.37 | Lu3 | 0.5 | 0.57 |
| 1961 | SBe | 30.7 | 2.60 | SBe | 30.7 | 2.60 | SBe | 39.9 | 2.60 | SBe | 34.8 | 2.60 |
| 1962 | B | 5.0 | 1.97 | B | 5.0 | 1.97 | B | 5.5 | 2.05 | B | 5.2 | 2.00 |
| 1963 | P | 16.3 | 0.98 | P | 16.8 | 1.00 | L1 | 10.3 | 3.41 | Lu1 | 3.3 | 1.23 |
| 1964 | R | 3.8 | 2.03 | R | 3.5 | 1.99 | L2 | 10.6 | 3.43 | Lu2 | 3.8 | L31 |
| 1965 | H | 12.1 | 3.52 | C | 33.4 | 3.04 | L3 | 20.4 | 3.71 | S | 3.9 | L32 |
| 1966 | SBe | 56.0 | 2.60 | SBe | 50.9 | 2.60 | SBe | 50.7 | 2.60 | SBe | 57.4 | 2.60 |
| 1967 | B | 4.8 | 1.94 | B | 4.5 | 1.89 | B | 5.8 | 2.09 | B | 5.8 | 2.09 |
| 1968 | P | 32.6 | 1.38 | P | 37.2 | 1.47 | L1 | 9.4 | 3.34 | S1 | 2.9 | 1.15 |
| 1969 | R | 3.8 | 2.03 | R | 3.4 | 1.97 | L2 | 14.2 | 3.61 | S2 | 7.8 | 1.71 |
| 1970 | H | 4.8 | 2.62 | C | 36.6 | 3.04 | L3 | 12.5 | 3.54 | S3 | 5.9 | 1.56 |
| 1971 | P | 60.1 | 1.74 | P | 56.9 | 1.72 | P | 64.1 | 1.78 | P | 65.3 | 1.79 |
| 1972 | W | 4.1 | 2.43 | W | 3.9 | 2.37 | W | 4.2 | 2.46 | W | 4.2 | 2.46 |
| 1973 | P | 51.2 | 1.66 | P | 52.1 | 1.67 | Ln1 | 2.7 | 1.96 | Lc1 | 2.5 | 1.87 |
| 1974 | B | 4.9 | 1.96 | B | 4.9 | 1.96 | Ln2 | 9.3 | 3.33 | Lc2 | 6.7 | 3.01 |
| 1975 | H | 4.8 | 2.62 | B | 1.7 | 1.14 | Ln3 | 7.1 | 3.07 | Lc3 | 6.0 | 2.88 |
| 1976 | W | 3.3 | 2.18 | W | 1.7 | 1.51 | W | 3.1 | 2.11 | W | 2.8 | 1.99 |
| 1977 | B | 2.5 | 1.41 | B | 2.2 | 1.31 | B | 2.8 | 1.50 | B | 3.3 | 1.63 |
| 1978 | B | 4.3 | 1.85 | F | 0.0 | 0.00 | Ln1 | 2.7 | 1.96 | Lc1 | 2.5 | 1.87 |
| 1979 | B | 4.3 | 1.85 | F | 0.0 | 0.00 | Ln2 | 9.3 | 3.33 | Lc2 | 6.7 | 3.01 |
| 1980 | O | 4.7 | 2.14 | O | 3.8 | 2.03 | Ln3 | 7.1 | 3.07 | Lc3 | 6.0 | 2.88 |
| 1981 | W | 5.9 | 2.86 | W | 5.8 | 2.84 | W | 7.3 | 3.10 | W | 6.8 | 3.02 |
| 1982 | B | 6.1 | 2.13 | B | 5.7 | 2.08 | B | 7.1 | 2.24 | B | 7.3 | 2.26 |
| 1983 | B | 4.8 | 1.94 | F | 0.0 | 0.00 | Ln1 | 2.7 | 1.96 | Lc1 | 2.5 | 1.87 |
| 1984 | B | 4.8 | 1.94 | F | 0.0 | 0.00 | Ln2 | 9.8 | 3.37 | Lc2 | 5.0 | 2.67 |
| 1985 | BE | 2.0 | 2.59 | BE | 2.0 | 2.59 | Ln3 | 10.3 | 3.41 | Lc3 | 9.4 | 3.34 |
| 1986 | W | 6.8 | 3.02 | W | 5.6 | 2.80 | W | 6.0 | 2.88 | W | 6.3 | 2.94 |
| 1987 | B | 5.0 | 1.97 | B | 4.7 | 1.92 | B | 5.8 | 2.09 | B | 5.8 | 2.09 |
| 1988 | B | 4.8 | 1.94 | F | 0.0 | 0.00 | Ln1 | 3.0 | 2.07 | Lc1 | 2.4 | 1.83 |
| 1989 | B | 4.8 | 1.94 | F | 0.0 | 0.00 | Ln2 | 8.0 | 3.19 | Lc2 | 3.7 | 2.31 |
| 1990 | BE | 2.0 | 2.59 | BE | 2.0 | 2.59 | Ln3 | 2.8 | 1.99 | Lc3 | 1.2 | 1.25 |
| 1991 | W | 8.0 | 3.19 | W | 7.5 | 3.13 | W | 9.3 | 3.33 | W | 9.0 | 3.30 |
| 1992 | R | 5.0 | 2.17 | R | 4.1 | 2.07 | R | 7.2 | 2.34 | R | 6.1 | 2.27 |
| 1993 | B | 4.8 | 1.94 | F | 0.0 | 0.00 | Ln1 | 10.3 | 3.41 | Lc1 | 4.4 | 2.52 |
| 1994 | B | 4.8 | 1.94 | F | 0.0 | 0.00 | Ln2 | 7.6 | 3.14 | Lc2 | 4.9 | 2.64 |
| 1995 | BE | 2.0 | 2.59 | BE | 2.0 | 2.59 | Ln3 | 4.0 | 2.40 | Lc3 | 4.4 | 2.52 |
| 1996 | W | 5.9 | 2.86 | W | 5.1 | 2.69 | W | 7.0 | 3.05 | W | 7.3 | 3.10 |
| 1997 | R | 3.9 | 2.04 | R | 3.3 | 1.96 | R | 4.9 | 2.16 | R | 5.3 | 2.20 |
| 1998 | R | 7.4 | 2.36 | R | 6.7 | 2.31 | Ln1 | 4.7 | 2.59 | Lc1 | 0.7 | 0.96 |
| 1999 | M | 6.9 | 2.06 | BE | 2.0 | 2.59 | Ln2 | 10.8 | 3.45 | Lc2 | 8.7 | 3.27 |
| 2000 | BE | 4.0 | 3.09 | M | 11.6 | 2.34 | Ln3 | 9.6 | 3.36 | Lc3 | 8.3 | 3.23 |
| 2001 | W | 3.4 | 2.21 | W | 2.3 | 1.79 | W | 5.8 | 2.84 | W | 5.5 | 2.78 |
| 2002 | R | 6.5 | 2.30 | R | 5.8 | 2.24 | R | 8.3 | 2.40 | R | 7.4 | 2.36 |
| 2003 | R | 5.8 | 2.24 | R | 4.4 | 2.10 | Ln1 | 5.6 | 2.80 | Lc1 | 1.0 | 1.14 |

P, potatoes: W, winter wheat: H, 1-year hay: K, kale: B, spring barley: SBe, sugar beet: R, winter rye: C, carrots: F, fallow: O, oats: BE, winter beans: M, maize: L1–L3, first, second and third year of a grazed grass+clover ley: Lu1–Lu3, first, second and third year of a lucerne ley: S1–S3, first, second and third year of a sainfoin ley: Ln1–Ln3, first, second and third year of a grass ley: Lc1–Lc3, first, second and third year of a grass+clover ley.

^a^AB treatment crops: potatoes, cereal, 1-year hay from 1938–75; barley, barley, beans (or oats) from 1978–95; rye, maize, beans since 1998.

^b^AF treatmenr crops: potatoes, cereal, root crop from 1938–75; fallow, fallow, beans from 1978–95; rye, beans, maize since 1998.

^c^LN3 treatment crop: 3-year grazed grass+clover leys with little N from 1938–70; 3-year grass leys with N since 1973.

^d^LC3 treatment crop: 3-year lucerne or sainfoin leys from 1938–70; 3-year grass+clover leys since 1973.

^e^Test crops are highlighted in grey.

^f^Yields of cereals (except maize) and beans are of grain at 85% dry matter; maize, grass, grass+clover, lucerne, sainfoin are whole crop at 100%

dry matter (leys usually cut twice a year); root crops are of fresh produce.

^g^Estimates of carbon inputs to the soil were calculated using protocols derived from those given by Bradbury *et al.* (1993).

**Table S2** Percentage of organic carbon in the top 25 cm of soils with and without FYM residues in different all-arable and ley–arable cropping systems since 1938; mean of five blocks. Woburn ley–arable experiment.

|  |  |  | Number of years since each block was phased in | | | | | | | | | | |
| --- | --- | --- | --- | --- | --- | --- | --- | --- | --- | --- | --- | --- | --- |
|  |  | 0 | 18 | 23 | 28 | 33 | 38 | 43 | 48 | 53 | 58 | 63 | 68 |
|  | FYM ^a^ residues | Years in which soils were sampled | | | | | | | | | | | |
| Rotation |  | 1938 | 1955–59 | 1960–64 | 1965–69 | 1970–74 | 1975–79 | 1980–84 | 1985–89 | 1990–94 | 1995–99 | 2000–04 | 2005–09 |
| AB | None | 0.98 | 0.97 | 0.94 | 0.95 | 1.03 | 0.93 | 0.88 | 0.93 | 0.91 | 0.87 | 0.96 | 0.90 |
|  | With | 0.98 | 1.05 | 1.06 | 1.04 | 1.09 | 1.01 | 0.95 | 0.97 | 0.96 | 0.92 | 1.00 | 0.91 |
| AF | None | 0.98 | 0.91 | 0.89 | 0.88 | 0.90 | 0.87 | 0.80 | 0.80 | 0.79 | 0.74 | 0.84 | 0.78 |
|  | With | 0.98 | 0.98 | 0.96 | 0.98 | 0.98 | 0.92 | 0.84 | 0.84 | 0.84 | 0.76 | 0.85 | 0.83 |
| LN3 | None | 0.98 | 1.08 | 1.08 | 1.13 | 1.23 | 1.12 | 1.13 | 1.09 | 1.17 | 1.09 | 1.28 | 1.21 |
|  | With | 0.98 | 1.19 | 1.26 | 1.32 | 1.40 | 1.25 | 1.26 | 1.29 | 1.28 | 1.17 | 1.33 | 1.32 |
| LC3 | None | 0.98 | 0.99 | 0.95 | 0.95 | 1.02 | 1.01 | 1.03 | 1.08 | 1.12 | 1.14 | 1.18 | 1.18 |
|  | With | 0.98 | 1.13 | 1.09 | 1.13 | 1.17 | 1.19 | 1.13 | 1.18 | 1.25 | 1.18 | 1.30 | 1.29 |
| Alternating rotations then long leys starting 1973–77 (1st cycle)^b^ | | | | | | | | | | | | | |
| LN8 | None | 0.98 | 1.01 | 0.97 | 0.99 | 1.05 | (1.03) | 1.07 | (1.07) | 1.25 | (1.07) | 1.43 | (1.27) |
|  | With | 0.98 | 1.12 | 1.11 | 1.15 | 1.12 | (1.15) | 1.20 | (1.18) | 1.33 | (1.26) | 1.50 | (1.34) |
| LC8 | None | 0.98 | 1.00 | 0.97 | 1.01 | 1.07 | (0.99) | 1.08 | (1.12) | 1.18 | (1.10) | 1.29 | (1.26) |
|  | With | 0.98 | 1.13 | 1.09 | 1.15 | 1.17 | (1.10) | 1.17 | (1.22) | 1.26 | (1.22) | 1.42 | (1.31) |
| Alternating rotations then long leys starting 1978–82 (2nd cycle)^c^ | | | | | | | | | | | | | |
| LN8 | None | 0.98 | 0.97 | 0.93 | 0.93 | 0.98 | 0.98 | (0.96) | 1.10 | (1.15) | 1.14 | (1.20) | 1.29 |
|  | With | 0.98 | 1.07 | 1.03 | 1.02 | 1.09 | 1.07 | (1.07) | 1.17 | (1.18) | 1.22 | (1.28) | 1.47 |
| LC8 | None | 0.98 | 0.95 | 1.01 | 0.96 | 1.02 | 0.99 | (1.00) | 1.11 | (1.15) | 1.12 | (1.25) | 1.31 |
|  | With | 0.98 | 1.08 | 1.16 | 1.19 | 1.21 | 1.15 | (1.15) | 1.22 | (1.34) | 1.24 | (1.40) | 1.55 |
| $\text{Rotation}\times\text{FYM}$ | S.E.D.^d^ | - | 0.070 | 0.064 | 0.065 | 0.073 | 0.073 | 0.073 | 0.08 | 0.088 | 0.079 | 0.083 | 0.104 |
|  | $\text{Rotation}\times\text{FYM}$ interaction all $F$ probabilities $> 0.05$ | | | | | | | | | | | | |

^a^ See footnotes to Table 1 for dates of FYM applications.

^b^ 1st cycle long leys were at the end of the eighth year of the 8–year ley when sampled in 1980–84, 1990–94 and 2000–04, data in brackets are at the end of the third year.

^c^ 2nd cycle long leys were at the end of the eighth year of the 8–year ley when sampled in 1985–89, 1995–99 and 2005–09, data in brackets are at the end of the third year.

^d^ Standard error of the differences of the means

**Figure S1** Schematic showing the flow of carbon through the Rothamsted turnover model (RothC-26.3) with rate constants and turnover times for the different carbon pools. Redrawn from Jenkinson (1990).

Plant material entering the soil is partitioned into two input compartments; decomposable plant material (DPM) and resistant plant material (RPM). Both DPM and RPM are decomposed in the soil by first-order processes to CO_2_, which is lost from the system, and to microbial biomass (BIO) and humified organic matter (HUM) which are both retained in the soil. Both BIO and HUM are decomposed further to give more CO_2_, biomass and humified matter. The soil is also assumed to contained a small (*c.*10 %) amount of inert organic material (IOM) which is inert to microbial decomposition, (at least in the short term).
